# Tumor delineation: The weakest link in the search for accuracy in radiotherapy

**DOI:** 10.4103/0971-6203.44472

**Published:** 2008

**Authors:** C. F. Njeh

**Affiliations:** Tyler Cancer Center (US oncology), Radiation Oncology Department, 910 East Houston Street, Tyler, Texas, 75702, USA

**Keywords:** Image-guided therapy, radiation therapy, tumor delineation

## Abstract

Radiotherapy is one of the most effective modalities for the treatment of cancer. However, there is a high degree of uncertainty associated with the target volume of most cancer sites. The sources of these uncertainties include, but are not limited to, the motion of the target, patient setup errors, patient movements, and the delineation of the target volume. Recently, many imaging techniques have been introduced to track the motion of tumors. The treatment delivery using these techniques is collectively called image-guided radiation therapy (IGRT). Ultimately, IGRT is only as good as the accuracy with which the target is known. There are reports of interobserver variability in tumor delineation across anatomical sites, but the widest ranges of variations have been reported for the delineation of head and neck tumors as well as esophageal and lung carcinomas. Significant interobserver variability in target delineation can be attributed to many factors including the impact of imaging and the influence of the observer (specialty, training, and personal bias). The visibility of the target can be greatly improved with the use of multimodality imaging by co-registration of CT with a second modality such as magnetic resonance imaging (MRI) and/or positron emission tomography. Also, continuous education, training, and cross-collaboration of the radiation oncologist with other specialties can reduce the degree of variability in tumor delineation.

## Introduction

**Figure 1:**
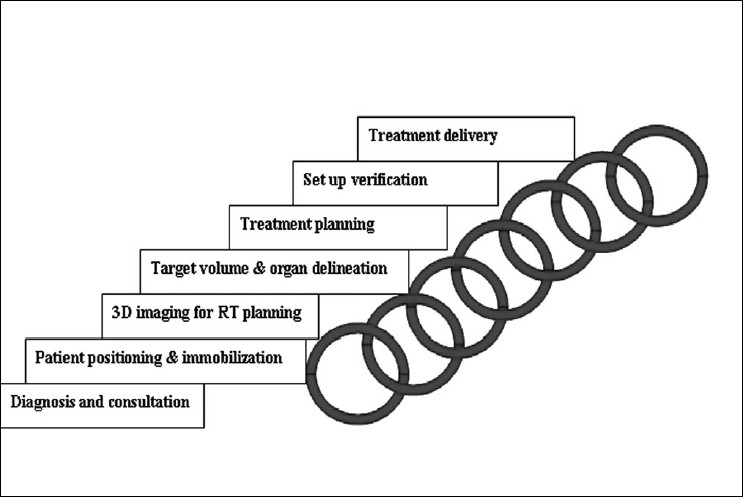
Some of the steps in radiotherapy that can be represented by links in a chain; treatment accuracy will be limited by the weakest link in the chain

Radiotherapy is one of the most effective modalities for the treatment of cancer. The fundamental tenet of radiotherapy is the delivery of a high dose to the tumor while limiting the dose to normal tissues. However, organs at risk and normal tissue tolerance have limited the amount of the dose that can be delivered to the tumor. With the advent of fast computers, multileaf collimation (MLC) and subsequently, the introduction of intensity-modulated radiation therapy (IMRT), conformal radiation therapy is now a reality (i.e., radiation is shaped to fit the 3D shape of the tumor). This makes it possible to escalate the dose to the tumor while limiting the dose to the normal tissues, with the possibility of providing better local control of the disease, enhancing the quality of life, and reducing treatment-associated morbidity. To achieve these benefits, we need to improve the accuracy of every step in treatment planning and delivery [Figure 1].

 As illustrated in Figure 1, radiation therapy is a long and complicated process; every step in the process is a potential source of error. Some of these uncertainties include: motion of the target, patient setup errors, patient movements, and delineation (contouring) of the target volume. These uncertainties can cause insufficient radiation dose coverage of the targeted tumor and an overdosage of normal tissues. Motion of the target can cause a geometric miss in the dose delivery. Hence, accurate radiation therapy involves knowing exactly where the tumor is at the time of treatment. As the famous Canadian Medical Physicist, Harold Johns once said, “if you can't see it you can't hit it and if you can't hit it you can't cure it”. The latest development of imaging techniques to monitor the target volume (IGRT) addresses that problem. However, the issue of target volume delineation has become less popular. Currently, a high degree of uncertainty is associated with the target volume of most cancer sites. IGRT is only as good as the accuracy with which the target is known. Hence, it is proposed here that the improvement in accuracy rendered by IGRT is limited by the accuracy of target delineation. Thus, this commentary will present the problem associated with target delineation, the merits and demerits of currently applied solutions, and future directions for research.

### Target delineation: The problem

Current practice in radiation therapy uses the definition of target volume proposed by the International Commission on Radiation Units and Measurements (ICRU).[     1] They proposed the following: gross tumor volume (GTV), clinical target volume (CTV), and planning target volume (PTV). The GTV is the part of the tumor that is visible with the use of 3D imaging so that the actual volume delineated is dependent on the imaging modality utilized and the data acquisition process. However, the clinically relevant volume is the CTV that includes the GTV as well as subclinical and microscopic anatomical spread patterns. However, these subclinical patterns are currently below the resolution limits of most modern imaging techniques. This problem is accounted for by adding margins around the GTV to generate the CTV. These margins are based on assumptions built from clinical or pathological experience and are subject to high degrees of uncertainty, making target delineation highly imprecise.

Unfortunately, the story does not end there—tumors can move throughout a treatment regimen. These displacements and deformations of the target may occur between fractions (referred to as interfraction) and/or during beam delivery (intrafraction). This motion of internal organs is due to physiological processes such as variations in the bladder or rectum filling, cardiac action, and respiration. The location of the target relative to the predetermined treatment isocenter may also change during treatment due to setup uncertainties. These issues have been reviewed extensively by a number of researchers including Langen and Jones,[2] Booth and Zavgorodni,[3] and Jaffray *et al*.[4] Target motion also affects treatment planning because the algorithms usually assume that the correct or at least the mean organ position as derived from the computed tomography (CT) imaging procedure, is reproduced throughout the treatment. In reality, a mobile organ is unlikely to be in its exact mean position at the time of imaging, causing the treatment to be planned with an organ offset from its assumed mean position. This introduces an extra ‘CT uncertainty’ into the treatment.[5] The traditional way to deal with or account for these uncertainties is by extending the CTV with an appropriate safety margin, generating the planning target volume (PTV). These margins are again, based on clinical experience even though theoretical margins based on the observed variations have been suggested by McKenzie *et al.*[6]. More often than not, the PTV includes a large amount of normal healthy tissue within the high dose volume, thus, limiting the total dose that can be delivered to the PTV.

Recently, to address the problem of organ motion, many imaging techniques have been introduced to track the motion of tumors. Treatment delivery using these techniques is collectively called image-guided radiation therapy (IGRT). Some of the most available methods include transabdominal ultrasound, electronic portal imaging devices (EPID), implanted markers within room mega voltage (MV) or Kilovoltage (KV) X-rays, and in-room CT such as the cone beam CT. Cone beam CT options are based on either an additional KV system or the use of megavolt radiations from a therapy source.[7] IGRT technologies provide volumetric imaging of both the targeted structures and the surrounding normal tissue and hence, provide patient-specific verification that the intention has been satisfied. With enhanced precision of treatment, other uncertainties such as target delineation errors become important. Ultimately, IGRT is only as good as the accuracy with which the target is known.

**Figure 2:**
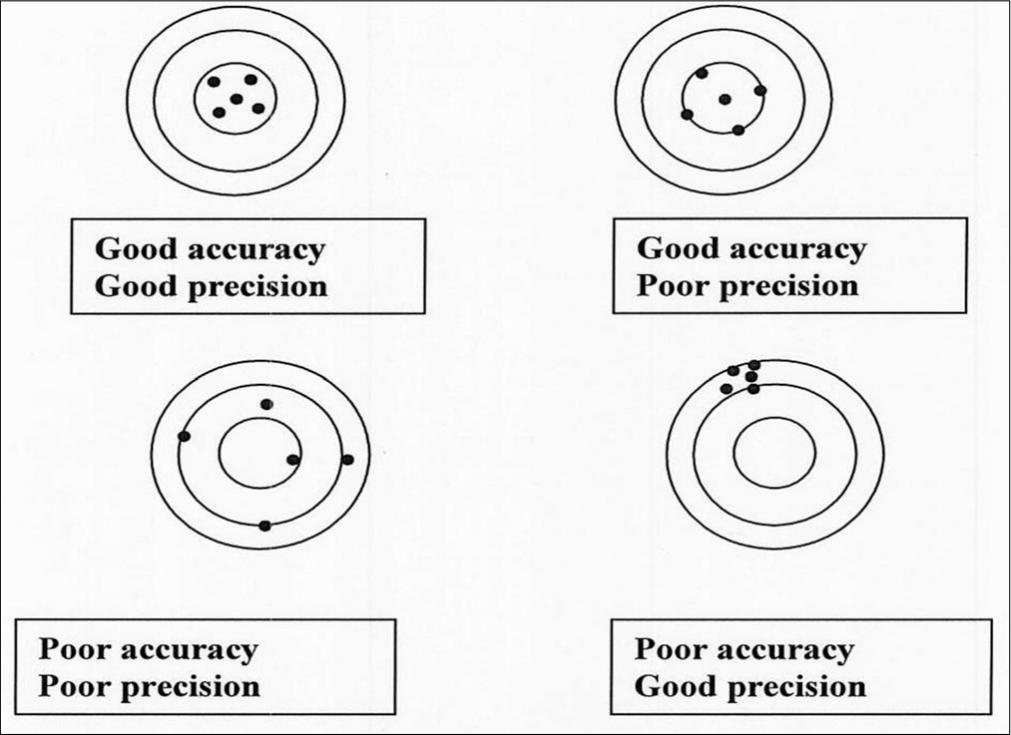
Illustration of the difference in precision and accuracy. The center of the circle represents the true value and the black dots represent the measured values (edited from Njeh and Langton with permission)

**Figure 3:**
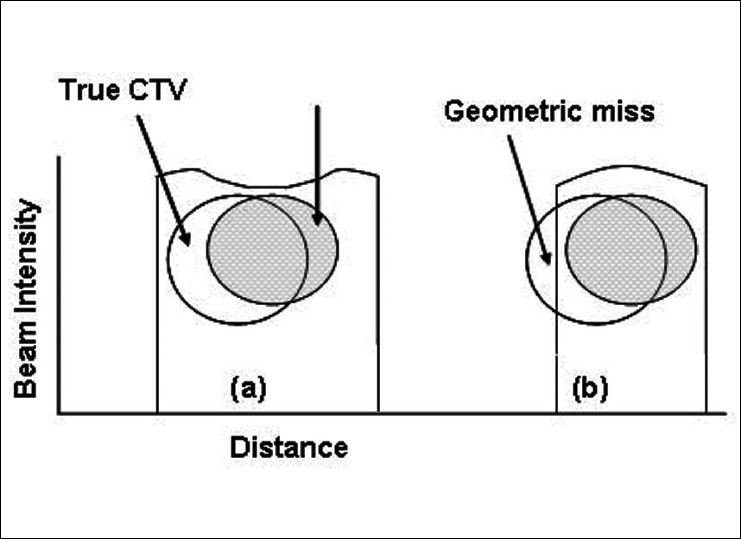
Illustration of the effect of high conformal radiation therapy and geometric miss due to delineation The shaded circle is the delineated CTV and the open circle is the true CTV. (a) Is the traditional 3D conventional RT with big margin and (b) Is the conformal RT with small margin and shows a portion of the tumor not covered by the prescribed dose

The previous sections have highlighted the problems with target definition and the applied solutions. However, another dimension to the problem exists which is the actual delineation (contouring) of the target volume. This is the missing or the weakest link in the search for accuracy in radiotherapy [Figure 1]. Errors in target contouring generate systematic errors which no level of image guidance will eliminate. This also brings home the difference in precision and accuracy. Proper contouring of the target volume improves accuracy whereas image guidance improves precision. In other words, you can consistently hit a wrong target (high precision but poor accuracy), but what is required is to consistently hit the right target (high precision, high accuracy) during the course of the radiation therapy [Figure 2]. Accuracy in tumor delineation cannot be overemphasized in this era of IMRT. This is because, with IMRT, there is a high gradient in dose fall-off; consequently, a geometric miss due to tumor delineation will result in a higher differential in dose delivered to the target [Figure 3].

### Evidence of tumor delineation variability

Target volume contouring is a major source of errors in most disease sites including nonsmall cell lung cancers, prostate, and head and neck external-beam radiation treatment. Delineation errors remain constant during the course of radiation therapy and therefore, have a large impact on the dose to the tumor. Major sources of tumor volume delineation variation are visibility of the target, including its extensions (impact of imaging protocol), disagreement on the target extension, and interpretation or lack of delineation protocols.[8,9]

The magnitude of the contouring problem is seen when studies of inter/intraobserver variability are conducted. There are not enough studies in the literature highlighting the level of inter- and intraobserver variability in target delineation. However, few researchers have demonstrated the variation in tumor volume delineation across physicians. These studies have been reviewed by Weiss and Hess.[9] They found that the variation in the ratio of maximum to minimum contoured volume for the prostate ranged between 1 and 1.6. The variation of the delineated prostate volumes in axial scans was highest at the top and bottom of the prostate. In a study where the volume of the seminal vesicles was analyzed separately, the authors found a higher variability (up to fourfold) for the contours of the seminal vesicles.[10]

Weiss and Hess[9] observed in their literature review that the widest range in interobserver variation was reported in the delineation of head and neck tumors as well as esophageal and lung carcinomas. The size of the largest GTV was more than eight times the size of the smallest volume. They concluded that, in general, interobserver variations in the delineated volume have to be considered even for well-circumscribed carcinomas such as prostate and cerebral tumors with variations of an average factor of 1.3 to 2.

Most studies evaluating inter/intraobserver variability have focused on the tumor. Delineation of the critical organs, however, also impacts the evaluation of the treatment plan. Saarnak and colleagues[11] found interobserver variations of 10% in the bladder and of 11% in the rectum. Differences in delineation among the observers in this study were attributed to unclear organ boundaries in the CT images. These variations have the potential to affect the dose-volume histograms of critical structures and normal tissue complication probabilities.

In this era of three-dimensional (3D) conformal radiation therapy, there is a drive towards reducing margins around CTV and increasing the tumor dose. However, if there is a significant variability in target delineation, the question arises as to whether it is prudent to reduce the margin.

### Causes of variability

It is apparent that there is a significant interobserver variability in tumor (target) delineation that can be attributed to many factors including the impact of imaging (imaging modality and the technique) and influence of the observer (specialty, training, and personal bias). The choice of an adequate imaging modality and technique, e.g., window level and contrast application, is of essential importance in target delineation. In most tumors, contrast material increases detectability and helps to define the borders of the malignancy.[9]

A far more complex factor is the variation between observers themselves. There are variable interpretations of the extent of microscopic involvement. In a review of the variation between specialties, it was found that radiation oncologists tend to delineate larger volumes than physicians from other specialties.[9] The reason for this is only speculative and could be because of their unconscious integration of geometric uncertainties or maybe, because of their limited radiological knowledge. After all, as Weiss and Hess[9] put it, “the contouring of a target volume is influenced to a large extent by the observer's subjective interpretation of what he or she sees on the images”. The training of the oncologists and instructions on contouring also have a significant impact on contouring variability. It has been reported that less experienced physicians contoured larger tumor volumes than experts.

### Solutions

The introduction of CT in the late 70s revolutionized radiation therapy, leading to significant improvements in CT data acquisition, processing, and display. Despite these developments in CT and better visibility of the tumors, a different interpretation of target extension remains a major source of error. High observer variability in CT-based definition of the GTV can still occur. Also, conventional CT has limitations in terms of distinguishing between benign and malignant tissues. The visibility of the target can be greatly improved with the use of multimodality imaging by co-registration of CT with a second modality such as magnetic resonance imaging (MRI) and/or positron emission tomography (PET).

Both in the head and neck and in the prostate, CT-MRI co-registration decreases the target volume and its variability. MRI has been claimed to be necessary for target volume definition of many tumors in radiation therapy planning because of its good depiction of soft tissue and the easy acquisition of multiplanar views.[12] One researcher found reductions of up to a factor of 3.5 in interobserver variability in prostate cancer delineation when MRI was used in conjunction with CT. CT-derived prostate volumes are larger than MR-derived volumes, especially toward the seminal vesicles and the apex of the prostate. Using MRI for delineation of the prostate reduces the amount of irradiated rectal wall, and could reduce rectal and urological complications.[13] It is thought that MRI of the prostate might lead to the “true” identification of the real anatomic prostate volume due to an improved visibility of the organ's boundaries.

CT does not seem to be suitable for distinguishing between tumor and nonmalignant structures such as blood vessels, noninvolved lymph nodes, and postobstructive inflammation and atelectasis (partial lung collapse) in lung cancers. Positron emission tomography (PET), on the other hand, is limited by poor spatial resolution that may make it difficult to accurately localize 2-(^18^ F)-fluoro-2-deoxy-glucose (FDG) uptake into an anatomic structure. Despite this limitation, FDG-PET has an accuracy of 85–100% of identifying pathologic lymph nodes. Furthermore, it has been suggested that FDG-PET can help differentiate between tumor and atelectasis.[14] The limitations of both PET and CT have been significantly reduced by combined PET-CT, a technique in which both PET and CT are performed sequentially during a single visit on a hybrid PET/CT scanner. However, some of the shortcomings of PET-CT have to be mentioned. Usually, the CT aspect of the PET-CT image is not of good quality compared to that of traditional CT. Also, the patient's position in the PET-CT is not the treatment position with immobilization. Hence, it is customary to fuse PET-CT images with another simulation CT in the correct treatment position. Nevertheless, CT-co-registers with PET images are promising for the delineation of lung cancer. Caldwell *et al.*[15] found a reduction in the interobserver variability when FDG PET images were co-registered with CT images for patients with nonsmall cell lung carcinoma. The mean ratios of largest to smallest GTV were 2.31 and 1.56 for CT only and for CT/PET co-registered data, respectively.[15] Therefore, a more consistent definition of the GTV can often be obtained if co-registered FDG-hybrid PET images are used. PET has its limitations including, but not limited to, poor spatial resolution (4–6 mm). Also, radiation oncologists may have limited expertise in the interpretation of FDG-PET images. If so, better training or the assistance of nuclear medicine specialists could improve the interpretation of FDG-PET by radiation oncologists.

This viewpoint of the implementation of multiple imaging modalities in the management of cancer has been reverberated by Schlegel,[16] the President of the European Federation of Organizations of Medical Physics (EFOMP). He suggests that to address the shortcomings of target delineation, “radiation oncology as a discipline needs to reinvent itself once more and pursue an ambitious development roadmap that will ultimately enable radiation oncologists and physicists to characterize the tumor in terms of the 3Ms.” The 3Ms, according to Schlegel,[16] represents morphology (anatomical structure), movement, and molecular (functional) profiling of the tumor.

Some researchers have identified the lack of continuous education and training as a cause of the variability in tumor delineation.[9] To address this problem, the American Society for Therapeutic Radiology and Oncology (ASTRO) introduced “e-countouring” sessions during the 2005 annual meeting, whereby attendees matched their contours with those of the experts. It has also been suggested that improved guidelines for tumor delineation increases the agreement between observers in prostate, lung, and nasopharyngeal tumors.[8] For example, Bowden *et al.*[17] found that applying a delineation protocol improved delineation accuracy. The average variation of the measured GTV was reduced from 20% without the protocol to 13% with the protocol. Their improved protocol included guidelines concerning level and window settings, and tumor identification by a diagnostic radiologist.

It is also recommended that radiation oncologists should collaborate with other specialties, such as radiologists. This view has been echoed by the Royal College of Radiologists in their 2004 guidance on the optimal imaging strategies for common cancers.[18] They recommended the development of closer links between radiologists and oncologists to optimize the interpretation of imaging and target volume definition. After all, radiologists are trained to read and interpret films and oncologists to treat cancer.

## Conclusion

It is evident that tumor delineation is currently the weakest link in radiotherapy accuracy and will continue to have a significant impact until improvement in tumor delineation is achieved. With the advancement of computer programming and imaging technology, especially functional imaging using PET, there is a possibility of converging and making tumor identification and definition less subjective and less observer-dependent.
